# A Bionic‐Homodimerization Strategy for Optimizing Modulators of Protein–Protein Interactions: From Statistical Mechanics Theory to Potential Clinical Translation

**DOI:** 10.1002/advs.202105179

**Published:** 2022-02-15

**Authors:** Jin Yan, Xiaoqiang Zheng, Weiming You, Wangxiao He, Guang‐Kui Xu

**Affiliations:** ^1^ Department of Tumor and Immunology in Precision Medical Institute and National & Local Joint Engineering Research Center of Biodiagnosis and Biotherapy The Second Affiliated Hospital of Xi'an Jiaotong University Xi'an 710004 China; ^2^ Institute for Stem Cell & Regenerative Medicine The Second Affiliated Hospital of Xi'an Jiaotong University Xi'an 710004 China; ^3^ Department of Medical Oncology and Department of Talent Highland The First Affiliated Hospital of Xi'an Jiaotong University Xi'an 710061 China; ^4^ Laboratory for Multiscale Mechanics and Medical Science SVL School of Aerospace Engineering Xi'an Jiaotong University Xi'an 710049 China

**Keywords:** bionic‐dimerization, nanomedicine, peptide, protein–protein interactions, statistical mechanics theory

## Abstract

Emerging protein–protein interaction (PPI) modulators have brought out exciting ability as therapeutics in human diseases, but its clinical translation has been greatly hampered by the limited affinity. Inspired by the homodimerize structure of antibody, the homodimerization contributes hugely to generating the optimized affinity is conjectured. Herein, a statistical‐mechanics‐theory‐guided method is established to quantize the affinity of ligands with different topologies through analyzing the change of enthalpy and the loss of translational and rotational entropies. A peptide modulator for p53‐MDM2 termed CPAP is used to homodimerize connecting, and this simple homodimerization can significantly increase the affinity. To realize the cellular internalization and tumor accumulation, ^Dimer^CPAP and ^Mono^CPAP are nanoengineered into gold(I)‐CPAP supermolecule by the aurophilic interaction‐driven self‐assembly. Nano‐^Dimer^CPAP potently suppressed tumor growth in lung cancer allograft model and a patient‐derived xenograft model in more action than Nano‐^Mono^CPAP, while keeping a favorable drug safety profile. This work not only presents a physico‐mechanical method for calculating the affinity of PPI modulators, but also provides a simple yet robust homodimerization strategy to optimize the affinity of PPI modulators.

## Graphical abstract

Inspired by the homodimerize structure of antibody that efficiently targeting extracellular PPIs, the homodimerization contributes hugely to generating the optimized affinity is conjectured. A de novo statistical‐mechanics‐theory‐guided method is established to quantize the affinity of ligands with different topologies through analyzing the change of enthalpy and the loss of translational and rotational entropies.

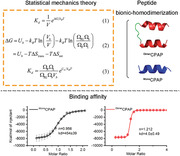

## 1 Introduction

Proteins are the basic building blocks of life that are typically controlled through 650 000 protein–protein interactions (PPI) in the human interactome.^[^ ^1^ ^]^ Emerging PPI modulators have brought out exciting ability as specific probes to regulate sophisticated biological signaling and vast potential as therapeutics to correct aberrant PPIs in great variety of human diseases including but not limited to: cancer, infectious diseases, and neurodegenerative diseases.^[^ ^2^ ^]^ In contrast to conventional enzyme and receptor targets, PPI targets always suffer from low affinity because of rather flat and non‐continuous protein surfaces without discrete binding pockets.^[^ ^3^ ^]^ To address it, antibody molecules were used to targeted extracellular PPIs, and some of them have been approved for application, such as Nivolumab for blocking PD‐1/PD‐L1 interaction and Ipilimumab for disturbing CTLA‐4/B7 interaction.^[^ ^4^ ^]^ Nevertheless, antibody molecules doesn't work inside the cell due to the loss of topological structure in response to the intracellular reducing environment.^[^ ^5^ ^]^ Although there have been some successes in strengthening associations between molecules and their specific targets using multivalency, the preparation of such molecules that can simultaneously bind multiple sites on targeted protein is always limited by the lack of binding sites on targeted protein and the insufficient quantity of effective ligands.^[^ ^6^ ^]^ Hence, it is urgent and meaningful to develop a general formula guidance to obtain high‐affinity intracellular PPI modulators.

Antibody, a class of homodimer immunoglobulin containing a pair of antigen‐binding domains, often possess high binding affinity with PPI targets at the level of pM.^[^ ^7^ ^]^ In theory, the binding affinity contributed by the change of binding enthalpy, the loss of translational entropy and the missing of rotational entropy.^[^ ^8^, ^9^ ^]^ As for antibody molecules, the physical connection between two antigen‐binding domains is a geometric constrain that would significantly decrease the rotational entropy, resulting in an increased affinity.^[^ ^9^ ^]^ Inspired by this naturally evolved topological structure of antibody, we hypothesized that this homodimerization presumably contributes hugely to generating the optimized binding affinity for peptide molecules that target intracellular PPIs.^[^ ^10^, ^11^, ^12^ ^]^ To verify it, we tried to calculate affinity contributions via atomistic molecular‐dynamics (MD) simulations.^[^ ^13^ ^]^ However, conventional MD methods can't satisfy the demand of differentiated topology,^[^ ^14^ ^]^ so we de novo established a statistical‐mechanics‐theory‐guided method to quantize the affinity of ligands with different topologies through comprehensively analyzed the change of binding enthalpy and the loss of translational as well as rotational entropy.^[^ ^8^, ^15^ ^]^

Herein, as a proof of concept, a peptide modulator for p53‐MDM2^[^ ^12^, ^16^ ^]^ termed CPAP was used to homodimerize connecting by a short and semiflexible linker consisting a 6‐aminohexanoic acid and two PEG. Comparatively calculating the binding affinity to MDM2 of CPAP dimer (^Dimer^CPAP) and CPAP monomer (^Mono^CPAP) through the established statistical‐mechanics method, we found that CPAP homodimerization could increase the affinity by two orders of magnitude. As expected, this theoretical result was proved by the affinity quantification measured by isothermal titration calorimetry (ITC), as evidence by a 128‐fold increment of MDM2 binding. In order to further verify the preponderance of dimerization and overcome the pharmacological hurdles of cellular internalization and tumor accumulation, ^Dimer^CPAP and ^Mono^CPAP were nanoengineered into gold(I)‐CPAP cluster termed Nano‐^Dimer^CPAP or Nano‐^Mono^CPAP through an aurophilic interaction‐driven self‐assembly among Au‐CPAP precursors [Au(I)‐S‐CPAP]_n_. Self‐evidently, Nano‐^Dimer^CPAP potently restored p53 signaling pathway in more action that Nano‐^Mono^CPAP. Moreover, the superiority of CPAP homodimerization in potency was systematically investigated in a LUAD allograft model and a LUAD‐patient‐derived xenograft (PDX) mice model. Taken together, this work not only presents a de novo physico‐mechanical method for calculating the affinity of PPI modulators with different topological structures, but also provides a universal and simple homodimerization strategy to optimize the binding affinity of intracellular PPI modulators.

## 2 Result and Discussion

**Figure 1:**
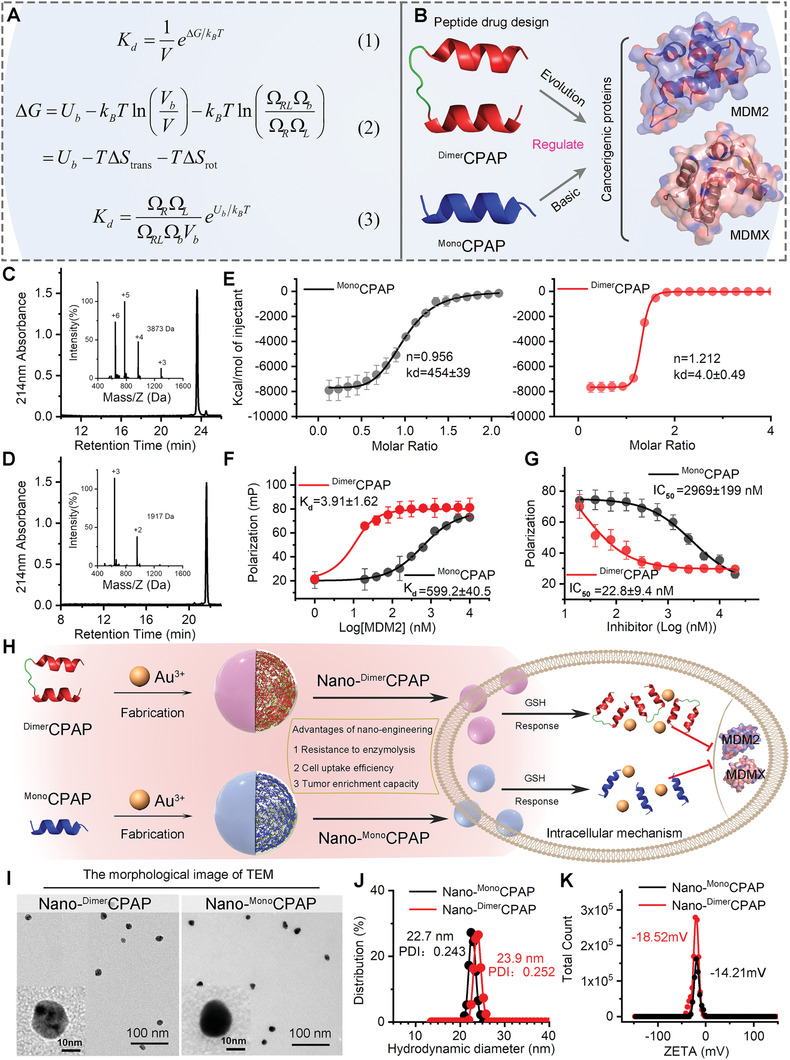
Statistical mechanics theory for binding affinity of dimeric strategy in the intervention of disease PPIs. A) Illustration of the statistical mechanics theory. B) The schematic diagram of function of ^Dimer^CPAP and ^Mono^CPAP. C,D) ^Dimer^CPAP and ^Mono^CPAP analyzed by HPLC and electrospray ionization mass spectrometry (ESI‐MS), which was performed on a reversed‐phase C18 column (Waters XBridge 3.5 µm, 4.6 × 150 mm) at 40 °C. E) Quantification of the interactions of ^syn^MDM2 with varying concentrations of ^Dimer^CPAP and ^Mono^CPAP by isothermal titration calorimetry (ITC). Each curve is the mean of 3 independent measurements at 25 °C in 10 × 10^−3^ m HEPES, 150 × 10^−3^ m NaCl, pH 7.4. F) Fluorescence polarization (Fp) binding assay of ^Dimer^CPAP or ^Mono^CPAP to MDM2 protein. G) Competitive FP‐based binding assay of ^Dimer^CPAP or ^Mono^CPAP to MDM2/p53 complex. H) Schematic depiction for nano‐engineering modification of ^Dimer^CPAP and ^Mono^CPAP. I) TEM images of Nano‐^Dimer^CPAP and Nano‐^Mono^CPAP. J) Hydrodynamic diameter and K) ZETA potential of Nano‐^Dimer^CPAP and Nano‐^Mono^CPAP measured in PBS buffer at pH 7.4.

We firstly established a statistical‐mechanics‐theory‐guided method to quantize the affinity of ligands with different topologies through comprehensively analyzed the change of binding enthalpy and the loss of translational as well as rotational entropy. Based on statistical mechanics,^[^ ^8^, ^15^ ^]^ one can derive the expression of the dissociation constant of a receptor‐ligand bond (see Supplementary Information)

(1) $$K_{d}=\frac{1}{V}e^{\Delta G/k_{B}T}$$

where *V* is the volume of the solution, Δ*G* represents the free energy difference upon the binding, *k*_B_ is the Boltzmann constant, and *T* is the temperature. The free energy difference Δ*G* can be given by (see Supplementary Information)

(2) $$\begin{array}{ccc} \Delta G & = & U_{b}-k_{B}T\ln\left(\frac{V_{b}}{V}\right)-k_{B}T\ln\left(\frac{\Omega_{\mathrm{RL}}\Omega_{b}}{\Omega_{R}\Omega_{L}}\right)\\ & = & U_{b}-T\Delta S_{\mathrm{trans}}-T\Delta S_{\mathrm{rot}} \end{array}$$

where *U*_b_ is the binding enthalpy of a receptor and a ligand, Δ*S*_trans_ = −*k*_B_ln (*V*_b_/*V*) is the loss of translational entropy upon the binding with *V*_b_ being the binding translational volume of the receptor and ligand, and Δ*S*_rot_ = −*k*_B_ln (Ω_RL_Ω_b_/Ω_R_Ω_L_) is the loss of rotational entropy upon the binding with Ω_RL_ being the rotational space volume of the receptor–ligand bond, Ω_b_ being the rotational space volume of the binding domain of the ligand with respect to the binding domain of the receptor in the bond, Ω_R_ and Ω_L_ being the rotational space volumes of a free receptor and ligand. Then, the dissociation constant can be rewritten as (**Figure** **1**A)

(3) $$K_{d}=\frac{\Omega_{R}\Omega_{L}}{\Omega_{\mathrm{RL}}\Omega_{b}V_{b}}e^{U_{b}/k_{B}T}$$

From Equation (3), one can see that the dissociation constant is proportional to the rotational phase space volumes of the receptor or ligand. Let $K_{d}^{\left(m\right)}$ and $K_{d}^{\left(d\right)}$ represent the dissociation constants of the monomer and the dimer, respectively. For the same receptors and ligands, the binding enthalpy *U*_b_ and the binding translational volume *V*_b_ would not change for the monomer and the dimer, which induces that their loss of translational entropies Δ*S*_trans_ are also the same. Therefore, the difference between $K_{d}^{\left(m\right)}$ and $K_{d}^{\left(d\right)}$ is determined by the loss of the rotational entropy Δ*S*_rot_, which is related to the rotational phase space volumes. Since the rotational phase space volume Ω_b_ would not vary for same proteins, Δ*S*_rot_ is determined by three other rotational phase space volumes (Ω_R_, Ω_L_, and Ω_RL_).

For the binding between the MDM2 and ^Mono^CPAP proteins, both of the receptor (i.e., MDM2) and the ligand (i.e., ^Mono^CPAP) can freely rotate, and one has $\Omega_{\mathrm{RL}}^{\left(m\right)}=\Omega_{R}^{\left(m\right)}=\Omega_{L}^{\left(m\right)}=\int \int \int \sin\;\theta d\theta d\varphi d\phi =8\pi^{2}$ with *θ*, *ϕ*, and *φ* being the Euler angles. The rotational phase spaces can be quantitatively described by these three Euler angles. For the binding between the MDM2 and ^Dimer^CPAP proteins, the MDM2 can still rotate freely, which will induce $\Omega_{\mathrm{RL}}^{\left(d\right)}=\Omega_{R}^{\left(d\right)}=8\pi^{2}$. However, since the linker is quite short (≈1 nm), there exist remarkable steric effects between two binding domains of the ^Dimer^CPAP, which will strongly constrain the free rotation of the binding domains of ^Dimer^CPAP. This means that the loss of the rotational entropy of ^Dimer^CPAP upon binding is much less than that of ^Mono^CPAP upon binding. Therefore, our theory shows that the affinity of the ^Dimer^CPAP will be greatly higher than that of ^Mono^CPAP. Based on the geometric size of the dimer, one may estimate the rotational motion differences (Δ*θ*, Δ*ϕ*, and Δ*φ*) in the constrained environment are in the range of π/3∼π/2. Then, we can obtain the rotational phase volume of the ligand $\Omega_{L}^{\left(d\right)}$ in the range of *π* ^2^/8∼*π* ^2^/18. From the above, one can naturally obtain the ratio $K_{d}^{\left(m\right)}/K_{d}^{\left(d\right)}$ between the dissociation constants of ^Mono^CPAP and ^Dimer^CPAP bound to MDM2 in the range of 64∼144. Based on these analyses, we show that the dissociation constant $K_{d}^{\left(d\right)}$ could be evidently decreased by linking the monomers to form a dimer, and their ratio can be up to two orders of magnitude. In our method, the same amount of CPAP proteins (i.e., the same amount of binding sites is used in experiments, meaning that the concentration of ^Dimer^CPAP is half of that of ^Mono^CPAP. Only due to the rotational constrain of the ligand in the dimer, our theory predicts that the affinity can be greatly improved, as shown in Equation (3).

To verify our theory, both ^Dimer^CPAP and ^Mono^CPAP were synthesized by the solid‐phase peptide synthesis of Fmoc and purified by reversed phase HPLC (Figure 1B).^[^ ^17^ ^]^ The corrected molecular weights of ^Dimer^CPAP and ^Mono^CPAP were identified by electrospray ionization mass spectrometry (ESI‐MS), and the over 95% purity was identified by Analytical high performance liquid chromatography (Figure 1C,D). More importantly, isothermal titration calorimetry (ITC) analysis revealed the association constants of the ^Dimer^CPAP and ^Mono^CPAP bound to MDM2 respectively by $K_{d}^{\left(d\right)}=\;4.0$ × 10^−9^ m (Figure 1E) and $K_{d}^{\left(m\right)}=\;454.0$ × 10^−9^ m (Figure 1E), leading to a quantitative relation $K_{d}^{\left(m\right)}=\;113.5\;K_{d}^{\left(d\right)}$, which is in excellent agreement with our theoretical predictions. Meanwhile, as shown in Figure S1 (Supporting Information), no binding constants were detected in other groups, such as buffer to buffer, ^Dimer^CPAP to buffer and ^Mono^CPAP to buffer. The result indicated that buffer system didn't influence the molecular binding affinity. At the same time, the binding affinity of ^Dimer^CPAP or ^Mono^CPAP was verified by Fluorescence polarization (Fp) assays, in which different concentrations of ^Dimer^CPAP‐FITC or ^Mono^CPAP‐FITC were incubated with MDM2 protein. And as shown in Figure 1F, the binding constants were respectively $K_{d}^{\left(d\right)}=\;3.9$ × 10^−9^ m and $K_{d}^{\left(m\right)}=\;599.2$ × 10^−6^ m. It can be concluded that the bind constant of the ^Dimer^CPAP bound to MDM2 exceeded two orders of magnitude than that of ^Mono^CPAP. Similarly, the inhibitory effects of CPAP on the interactions between p53 and MDM2 were measured by Fp‐based competition assays, where different concentrations of CPAP were preincubated with MDM2/p53‐FITC complexes. As shown in Figure 1G, the half‐maximal inhibitory concentrations (IC_50_ values) of ^Dimer^CPAP were obviously less than that of or ^Mono^CPAP, indicating the stronger affinity of ^Dimer^CPAP. The tendency of above data was consistent with the theoretical simulation prediction. These results demonstrated that the affinity of the monomers could be significantly promoted by physically connecting them to be dimers through a short linker.

Furthermore, to alleviate the pharmacological hurdles of cellular internalization and tumor accumulation, both ^Dimer^CPAP and ^Mono^CPAP were nanoengineered into a spherical nanostructure via a aurophilicity‐driven self‐assembly among Au(I)‐peptide mercaptan precursors (Figure 1G).^[^ ^11^, ^18^ ^]^ The well‐defined nanostructure was proved by the transmission electron microscopy (TEM) images, in which Nano‐^Dimer^CPAP and Nano‐^Mono^CPAP presented a spherical morphology with ≈20 nm diameter (Figure 1H). In line with TEM images, Nano‐^Dimer^CPAP possessed the similar hydrodynamic diameter by about 20 nm with Nano‐^Mono^CPAP, indicative of the similar hydrodynamic characteristics of the two spherical supramolecule (Figure 1I). Moreover, the Zeta potential of Nano‐^Dimer^CPAP and Nano‐^Mono^CPAP were respectively −18.52 and −14.21 mV (Figure 1J), suggesting the approximate surface charge and the subsequent similar colloidal stability. These results compelled us comparatively explored the cellular internalization and tumor accumulation of Nano‐^Dimer^CPAP and Nano‐^Mono^CPAP. As expected, both supramolecules presented the similar and satisfactory cellular internalization of cancer cells in sharp contrast to the two monomolecular peptides (Figure S2, Supporting Information). In addition, Nano‐^Dimer^CPAP and Nano‐^Mono^CPAP showed the approximately same amount of accumulation at tumor sites (Figure S3, Supporting Information), whereas neither ^Dimer^CPAP nor ^Mono^CPAP showed the obvious neoplastic accumulation (Figure S4, Supporting Information). Collectively, the results mentioned above revealed that both ^Dimer^CPAP and ^Mono^CPAP were nanoengineered into spherical supramolecule with the similar physicochemical and pharmaceutical properties.

**Figure 2:**
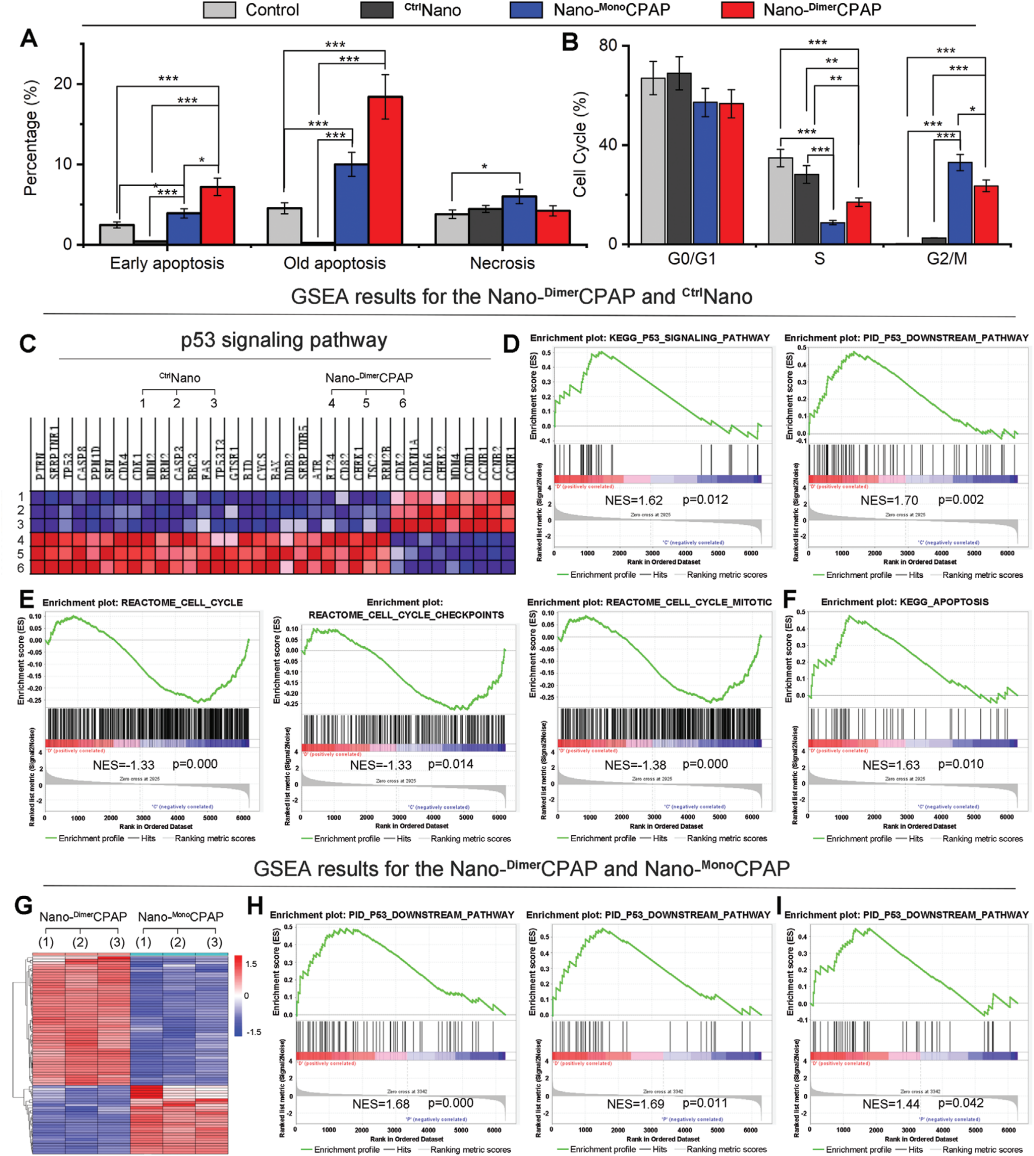
Nano‐^Dimer^CPAP potently activated p53 signaling cascades beyond Nano‐^Mono^CPAP in vitro. A) Apoptosis and necrosis analysis of NCI‐1650 cells incubated with PBS, Nano‐^Dimer^CPAP (10 µg mL^−1^), Nano‐^Mono^CPAP (10 µg mL^−1^) or ^Ctrl^Nano via flow cytometry for 48 h (*n* = 3, mean ± sd). B) Cell cycle analysis of NCI‐1650 cells treated with Nano‐^Dimer^CPAP, Nano‐^Mono^CPAP, ^Ctrl^Nano or PBS control for 48 h by FACS (*n* = 3, mean ± sd). C) Heat map of RNA‐Seq analysis of NCI‐H1650 cells’ mRNAs which were differentially expressed between Nano‐^Dimer^CPAP and ^Ctrl^Nano (*n* = 3). D) GSEA results for the p53 signaling pathway and the p53 downstream pathway. GSEA results for the REACTOME cell cycle checkpoints, E) the REACTOME cell cycle mitotic and F) the KEGG apoptosis. G) Hierarchical clustering of genes differentially expressed in NCI‐H1650 cells after exposure to Nano‐^Dimer^CPAP for 24 h compared with Nano‐^Mono^CPAP (*n* = 3). H) GSEA analysis of Nano‐^Dimer^CPAP versus Nano‐^Mono^CPAP showing the increased p53 signaling and downstream pathway. I) GSEA showing that the apoptosis of Nano‐^Dimer^CPAP is superior to Nano‐^Mono^CPAP.

As our hypothesis, the enhanced affinity contributed from homodimerization would result in the significantly increased potency. To verify it, we comparative investigated the in vitro potency of Nano‐^Dimer^CPAP and Nano‐^Mono^CPAP in NCI‐H1650 lung adenocarcinoma cell line through cell apoptosis and cell cycle analysis by flow cytometry. Expectedly, neither PBS nor ^Ctrl^Nano had obviously caused cancer cells apoptosis and cycle arrest (**Figure** **2**A,B and Table S1, Supporting Information). However, Nano‐^Dimer^CPAP significantly induced cancer cells apoptosis and cycle arrest in more action than Nano‐^Mono^CPAP. Meanwhile random mechanical damage may result in cell necrosis. As shown in Figure 2A, the proportion of necrosis of four groups was within the normal range, which was low as to be below 7.5%. For necrosis, there was no statistical difference between Nano‐^Dimer^CPAP and Nano‐^Mono^CPAP. For further mechanism exploration, proteome analysis was performed following a 24‐h incubation with PBS, Nano‐^Dimer^CPAP or Nano‐^Mono^CPAP. As shown in Figure 2C, Nano‐^Dimer^CPAP treatment aroused a noticeable change in p53 signaling pathway comparing with mock treatment. More importantly, gene set enrichment analysis (GSEA) revealed that the top changed pathways in response to Nano‐^Dimer^CPAP treatment involved in the up‐regulated p53 as well as its downstream signaling pathway (Figure 2D), down‐regulated cell cycle pathways (Figure 2E), and apoptosis pathways (Figure 2F). With the Comparison between Nano‐^Dimer^CPAP and Nano‐^Mono^CPAP treatment, 98 differential proteins can be found (Figure 2G and Figure S5, Supporting Information), and most of them enriched in p53 signaling pathway (Figure 2H), p53 downstream signaling pathway (Figure 2H), and apoptosis pathways (Figure 2I), suggesting the enhanced p53 activity in effect of Nano‐^Dimer^CPAP treatment. Meanwhile, similar results can be found that the strongest activation treatment was Nano‐^Dimer^CPAP group (Figure S6, Supporting Information). Collectively, these data convincingly validate that Nano‐^Dimer^CPAP possesses higher reactivated efficiency than Nano‐^Mono^CPAP in vitro via p53 restoration.

**Figure 3:**
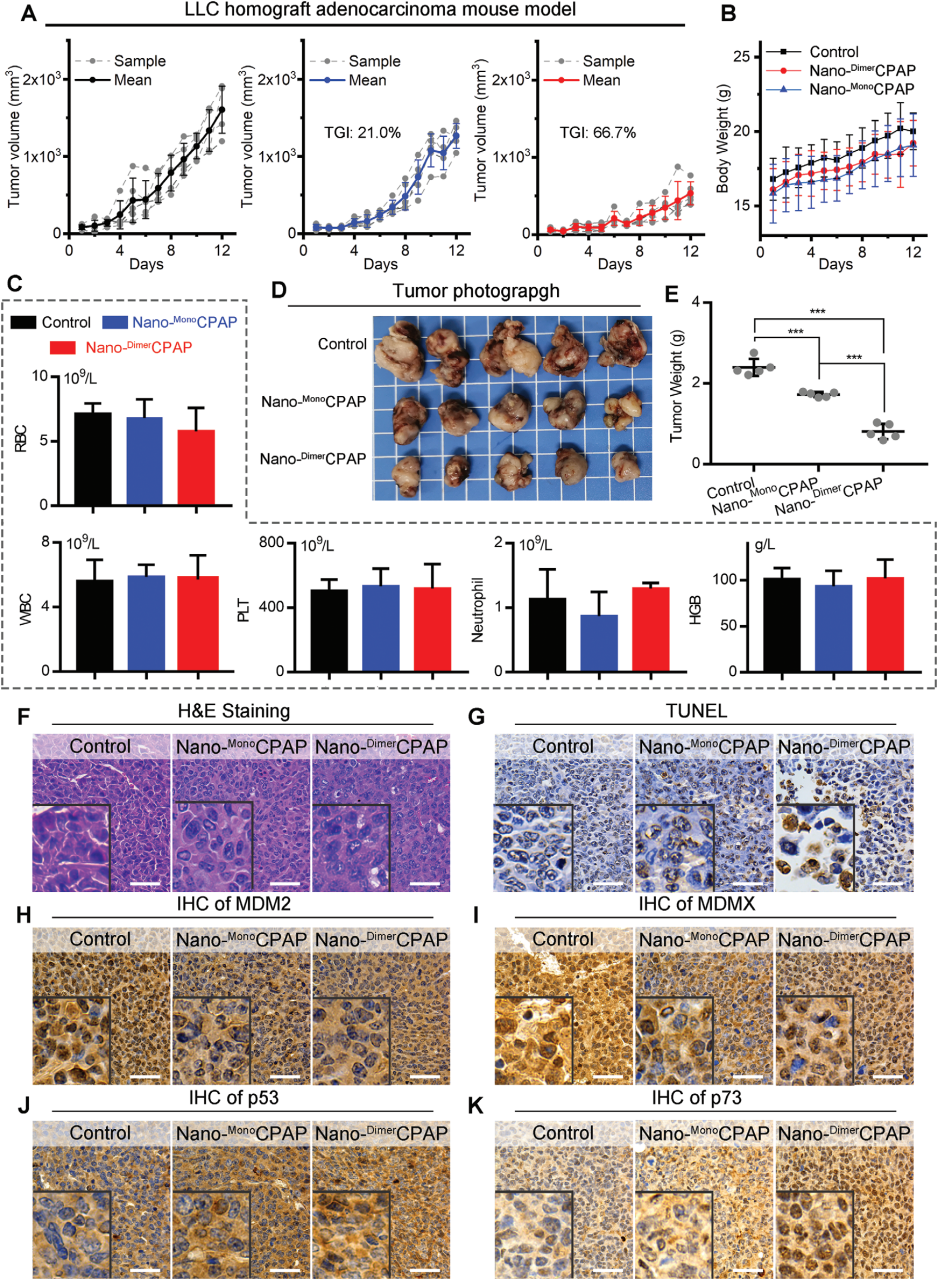
Nano‐^Dimer^CPAP potently suppressed tumor growth superior to Nano‐^Mono^CPAP in C57/B6 mice LUAD allograft model. A) Growth curves of LLC homograft model in C57/B6 mice with treatments, following the administration of control (PBS), Nano‐^Dimer^CPAP (2.5 mg kg^−1^) and Nano‐^Mono^CPAP (2.5 mg kg^−1^) (*n* = 5). B) Body weight of mice with the indicated treatments. C) Analysis of red blood cell (RBC), white blood cell (WBC), platelets (PLT), neutrophil, hemoglobin (HGB) in mice whole blood after treatments. D) Representative photographs and E) weight of tumor tissue isolated at the end of experiment. F,G) Representative images of H&E and TUNEL staining in tumor section from mice (scale bar: 50 µm). The immunohistochemical (IHC) staining for H) MDM2, I) MDMX, J) p53, and K) p73 in tumor sections from mice (scale bar: 50 µm).

To further compare the therapeutic efficacy of Nano‐^Dimer^CPAP to Nano‐^Mono^CPAP, an subcutaneous transplantation model of murine LUAD was established, in which 1 × 10^6^ LLC cells were subcutaneously inoculated into the fossa iliaca of C57BL/6 mouse. 10 days following the transplantation, mice bearing LLC tumor were randomly divided into three groups (*n* = 5 per group) to receive a 12‐day regimen of intravenous injection each other day as follows: Nano‐^Dimer^CPAP (2.5 mg kg^−1^), Nano‐^Mono^CPAP(2.5 mg kg^−1^), and normal saline (Control). As expected, both Nano‐^Dimer^CPAP and Nano‐^Mono^CPAP groups displayed therapeutic effects of tumor suppression in comparison to the PBS control (**Figure** **3**A), while maintaining a favorable safety as evidence by the body weights (Figure 3B) and pathological sections (Figure S7, Supporting Information). Furthermore, this therapeutic safety was approved again by the count analysis of red blood cells, white blood cells (leukocytes), platelets, neutrophils and hemoglobin (Figure 3C). More importantly, Nano‐^Dimer^CPAP inhibited tumor growth by 66.7%, better than the 21.0% inhibition value of Nano‐^Mono^CPAP (Figure 3A). ^Ctrl^Nano group had no obvious ability of significantly inhibiting tumor growth, comparing with corresponding Control (Figure S8, Supporting Information). Additionally, the average tumor weight of Nano‐^Mono^CPAP‐treated mice was threefold than that of Nano‐^Dimer^CPAP, supporting again the superiority of homologous dimerization (Figure 3D,E). To further compare the in vivo antitumor activity of Nano‐^Dimer^CPAP and Nano‐^Mono^CPAP at the histopathological level, we analyzed tumor tissues using hematoxylin and eosin (H&E) staining and terminal deoxynucleotidyl transferase‐mediated dUTP nick end labeling (TUNEL) techniques. As shown in Figure 3F,G, Nano‐^Dimer^CPAP significantly increased levels of apoptosis within tumor in comparison to Nano‐^Mono^CPAP groups. Furthermore, to explore the mechanism, we carried out the immunohistochemical staining to investigate the MDM2, MDMX, p53, and p73 levels in paraffin sections of tumor tissue. Both Nano‐^Dimer^CPAP and Nano‐^Mono^CPAP remarkably down‐regulated the levels of MDM2 (Figure 3H) and MDMX (Figure 3I) in the nucleus of tumor cell, resulting in the restoration of p53 (Figure 3J) and p73 (Figure 3K). Of note, Nano‐^Dimer^CPAP was more active than Nano‐^Mono^CPAP in these protein regulations (Figure 3H–K), providing additional evidence for the superiority of homodimerization. In short, Nano‐^Dimer^CPAP effectively and safely suppressed lung carcinoma progression by inhibiting MDM2/MDMX and activating p53/p73, while superior to Nano‐^Mono^CPAP group.

**Figure 4:**
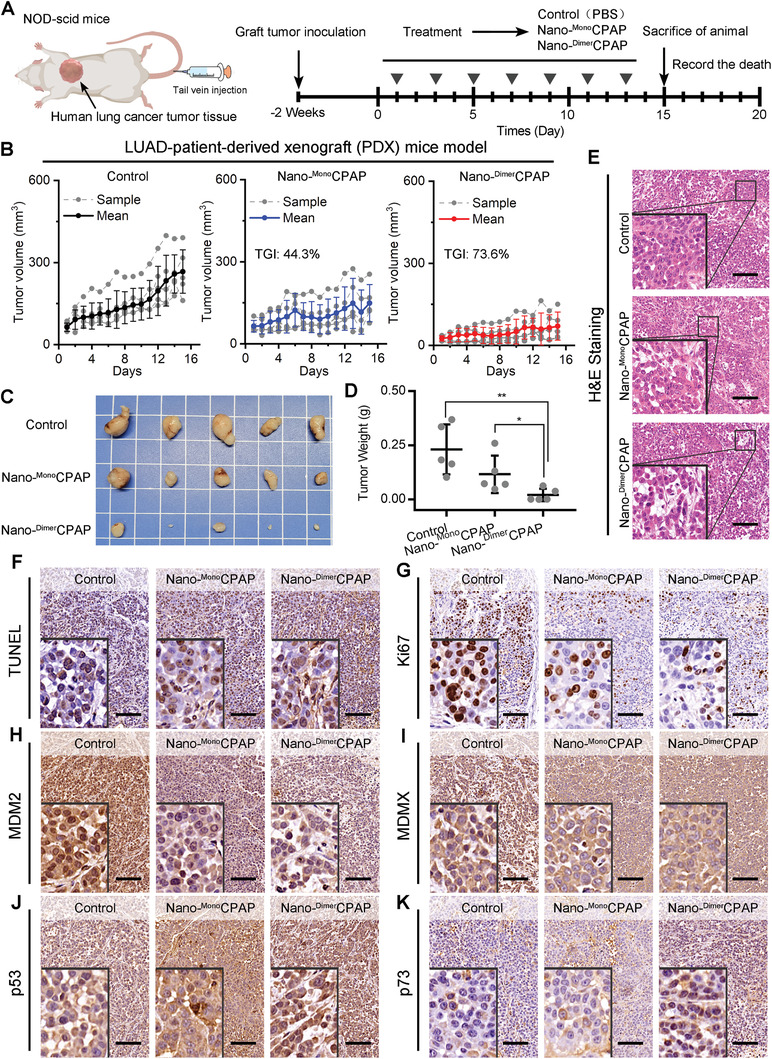
Nano‐^Dimer^CPAP enhanced more anticancer activity in LUAD patient‐derived tumor xenograft in NOD/SCID mice. A) Diagrammatic sketch of LUAD‐PDX mice model with indicated treatments. B) Growth curves of LUAD‐PDX mice model after administration of control (PBS), Nano‐^Dimer^CPAP (2.5 mg kg^−1^) and Nano‐^Mono^CPAP (2.5 mg kg^−1^) (mean ± sd, *n* = 5 per group). C) Images and D) weights of tumors excised at the end of treatment. *p*‐values were calculated by *t* test (*p < 0.05; **p < 0.01; ***p < 0.001). E) The H&E and F) TUNEL staining in tumor from mice after indicated treatments (scale bar: 200 µm). Representative images of IHC staining of G) Ki67, H) MDM2, I) MDMX, J) p53, and K) p73 in tumor section from mice with indicated treatments (scale bar: 200 µm).

For further verification, a patient‐derived xenograft (PDX) model of lung adenocarcinoma was established, which was more robustly recapitulated parental tumor molecular and biological features. To investigate the effectiveness of Dimer or Mono strategy, we comparatively tested respective effects on NOD/SCID mice bearing the surgically resected tumor from a LUAD‐patient. When the volume of tumor grows to 50 ± 25 mm^3^, mice were randomly assigned to respectively receive intravenous injection of PBS (control), Nano‐^Dimer^CPAP (2.5 mg kg^−1^) or Nano‐^Mono^CPAP (2.5 mg kg^−1^). During the 15‐day administration, the detail of schedule was shown as **Figure** **4**A. Of note, the volume of tumor analysis demonstrated that Nano‐^Dimer^CPAP treatment was the most efficacious at the end, yielding a tumor growth inhibition (TGI) rate of 73.6% (Figure 4B). The Nano‐^Mono^CPAP treatment was less effective than Nano‐^Dimer^CPAP treatment, which TGI rate is 44.3%. It can be inferred that Nano‐^Dimer^CPAP was nearly 1.7 times more potent than Nano‐^Mono^CPAP in tumor suppression. The tendency of tumor suppression was in line with data from tumor photos (Figure 4C), tumor weight (Figure 4D), and histological H&E staining images (Figure 4E). Furthermore, these results were proved again by the TdT‐mediated dUTP nick‐end labeling (TUNEL) staining (Figure 4F) and the immunohistochemical staining of Ki67 (Figure 4G). Comparing with PBS treatment, ^Ctrl^Nano cannot cause significant apoptosis in tumor tissue (Figure S9, Supporting Information). The result of H&E and TUNEL suggested that ^Ctrl^Nano lacked the ability to suppress tumor growth (Figure S9, Supporting Information). In quest of the mechanism of the tumor suppression in above LUAD‐PDX mice model, we explore MDM2, MDMX, p53, and p73 levels using immunohistochemical staining in paraffin sections of tumor tissue. As shown in Figure 4H,I, Nano‐^Dimer^CPAP dramatically down‐regulated levels of MDM2 and MDMX in the nuclei of tumor cells comparing with Nano‐^Mono^CPAP and control. As a result, the restoration of p53 and p73 further confirmed the tumor suppression ability and mechanism of Nano‐^Dimer^CPAP and Nano‐^Mono^CPAP (Figure 4J,K). The above results indicated that Nano‐^Dimer^CPAP was superior to Nano‐^Mono^CPAP. Moreover, there was no significant decrease for mice body weight (Figure S10, Supporting Information), suggesting the safety and no cytotoxicity of Nano‐^Dimer^CPAP and Nano‐^Mono^CPAP. In the meanwhile, the safety of Nano‐^Dimer^CPAP and Nano‐^Mono^CPAP were validated in detail by H&E staining of liver, kidney, spleen, lung, and heart (Figure S11, Supporting Information). In conclusion, Nano‐^Dimer^CPAP has a notable advantage over Nano‐^Mono^CPAP for clinical translation.

## 3 Conclusion

In this work, inspired by the homodimerization structure of antibody, we hypothesized that this homodimerization presumably contributes hugely to generating the optimized binding affinity for molecules that target intracellular PPIs. From theoretical perspective, a de novo statistical‐mechanics‐guided method was established to quantize the affinity of ligands with different topologies through comprehensively analyzed the change of binding enthalpy and the loss of translational as well as rotational entropy. As a proof of concept, we comparatively calculated the binding affinity to MDM2 of CPAP dimer (DimerCPAP) and CPAP monomer (MonoCPAP) through the established statistical‐mechanics method, and found that CPAP homodimerization could increase the affinity by two orders of magnitude, which was proved by the affinity quantification measured by isothermal titration calorimetry (ITC). Next, exploiting the aurophilic interaction‐driven self‐assembly, ^Dimer^CPAP and ^Mono^CPAP were nanoengineered into gold(I)‐CPAP supermolecule to overcome the pharmacological hurdles of cellular internalization and tumor accumulation. As expected, Nano‐^Dimer^CPAP potently restored p53 signaling pathway in more action that Nano‐^Mono^CPAP. Moreover, the superiority of CPAP homodimerization in potency was fully validated in a LUAD allograft model and a LUAD‐patient‐derived xenograft (PDX) mice model, while keeping a favorable drug safety profile. Taken together, this work not only presents a de novo physico‐mechanical method for calculating the affinity of PPI modulators with different topological structures, but also provides a universal and simple homodimerization strategy to optimize the binding affinity of intracellular PPI modulators.

## Conflict of Interest

The authors declare no conflict of interest.

## Supporting information

Supporting Information

## Data Availability

The data that support the findings of this study are available from the corresponding author upon reasonable request.
