# Supplementary material for: A Bionic‐Homodimerization Strategy for Optimizing Modulators of Protein–Protein Interactions: From Statistical Mechanics Theory to Potential Clinical Translation
Source: Adv Sci (Weinh). 2022 Feb 15;9(11):2105179. doi: 10.1002/advs.202105179 (PMC9008432; doi:10.1002/advs.202105179)
Supplement: Supplementary file 1 — Supporting Information [file ADVS-9-2105179-s001.pdf]

ADVANCED SCIENCE
Open Access

Supporting Information

for *Adv. Sci.*, DOI: 10.1002/advs.202105179

A Bionic-Homodimerization Strategy for Optimizing Modulators of Protein-Protein Interactions: from Statistical Mechanics Theory to Potential Clinical Translation

JinYan, Xiaoqiang Zheng, Weiming You, Wangxiao He* and Guang-Kui Xu**

Supplementary

A Bionic-Homodimerization Strategy for Optimizing Modulators of Protein-Protein Interactions: from Statistical Mechanics Theory to Potential Clinical Translation

Jin Yan[1,*], Xiaoqiang Zheng[2], Weiming You[1], Wangxiao He[3,2*]
and Guang-Kui Xu[4,*]

1. Department of Tumor and Immunology in precision medical institute and National & Local Joint Engineering Research Center of Biodiagnosis and Biotherapy, The Second Affiliated Hospital of Xi’an Jiaotong University, Xi’an 710004, China.
2. Institute for Stem Cell & Regenerative Medicine, The Second Affiliated Hospital of Xi’an Jiaotong University, Xi’an 710004, China.
3.Department of Medical Oncology and Department of Talent Highland, The First Affiliated Hospital of Xi’an Jiaotong University, Xi’an 710061, China.
4. Laboratory for Multiscale Mechanics and Medical Science, SVL, School of Aerospace Engineering, Xi'an Jiaotong University, Xi'an 710049, China.

[*] Corresponding authors:
Email: yanjin19920602@xjtu.edu.cn (J. Yan);
Email: hewangxiao5366@xjtu.edu.cn (W. He);
Email: guangkuixu@mail.xjtu.edu.cn (G. K. Xu).

1. Experimental Section

General remarks

All synthetic peptide sources were obtained from CS Bio (Shanghai) Ltd. All other chemicals used in this study were purchased from Sigma-Aldrich unless otherwise specified. Acetonitrile and water (HPLC grade) were purchased from Fisher Scientific Ltd. All products were used as received without further purification.

Synthesis of ^{Mono}CPAP or ^{Dimer}CPAP

All peptides were synthesized on appropriate resins on an CS bio 336X automated peptide synthesizer using the optimized HBTU activation/DIEA in situ neutralization protocol developed by an HBTU/HOBt protocol for Fmoc-chemistry SPPS. After cleavage and deprotection in a reagent cocktail containing 88% TFA, 5% phenol, 5% H_2O and 2%TIPS, crude products were precipitated with cold ether and purified to homogeneity by preparative C18 reversed-phase HPLC. The molecular masses were ascertained by electrospray ionization mass spectrometry (ESI-MS).

The characterization of ^{Mono}CPAP and ^{Dimer}CPAP via ITC

ITC measurements were performed in a Microcal 2000 calorimeter (GE Healthcare) at 25 °C in PBS, pH 7.4. Titrations were carried out by 20 stepwise injections, 2 μL at a time, of 200 μM peptide in the syringe to 20 μM MDM2 protein in the cell. Data were analyzed using the Microcal Origin program. Data points at saturation were used to calculate a mean baseline value, which was then subtracted from each data point.

Fluorescence Polarization (FP)-Based Binding and Competitive Binding Assay

To perform the FP binding assay, fluorescein isothiocyanate (FITC) was conjugated to MDM2 via its N-terminal amino group. To perform the FP competitive binding assay, fluorescein isothiocyanate (FITC) was conjugated to $^{15-29}$p53 via its N-terminal amino group. The resultant products MDM2-FITC or $^{15-29}$p53-FITC was HPLC-purified and lyophilized. MDM2 was synthesized and purified as previously described.[1] Fluorescence polarization-based binding assay was then performed as described previously [2], and the readings were taken using a fluorescence microplate reader (Tecan M2000, λex = 470 nm; λem = 530 nm.). ^{Mono}CPAP or ^{Dimer}CPAP peptide was serially 2-fold diluted in 10 mM Tris-HCl buffer (pH 7.0) containing 150 mM NaCl and 1 mM EDTA, and subsequently incubated with 200 nM MDM2-FITC or $^{15-29}$p53-FITC/MDM2 for 2 h in a total volume of 150 μL per well. Kd and IC_{50} values were calculated by nonlinear regression as described previously.[1]

Synthesis of Nano-^{Mono}CPAP or Nano-^{Dimer}CPAP

First, 2 mg of ^{Mono}CPAP or ^{Dimer}CPAP peptide was completely dissolved in a solution containing 500μL ethanol and 1.25mL ddH_20. After that, an aqueous solution of tetrachloroauric acid ($HAuCl_4 \cdot XH_2O$, 1 mL, 10 mM) was mixed with 500μL NH_2-PEG-SH (MW: 2000, 4 mg/ml in deionized water) and 2.25 ml HEPES (100mM, pH 7.0). Then it mixed with the prepared solution containing 2.25 ml deionized water and 2.25ml HEPES (100mM pH 7.0), sonicate for 10 min. Finally, removed the excess reactants by dialysis tubing (cutoff, 10 KDa) and washed twice by distilled water. Finally, we obtained Nano-^{Mono}CPAP and Nano-^{Dimer}CPAP.

Physicochemical properties of Nano-^{Mono}CPAP or Nano-^{Dimer}CPAP

The morphology and lattice structure were observed on high-resolution transmission electron microscopy (HRTEM), which was performed on an Talos F200X. One portion of the pellet was placed onto a carbon-coated copper grid for imaging with high-resolution transmission electron microscopy (HRTEM). The hydrodynamic size distribution was obtained from the dynamic light scattering (DLS) measurement (Malvern Zetasizer Nano ZS system). For Zeta potential measurement, Nano-^{Mono}CPAP or Nano-^{Dimer}CPAP was incubated with PBS at pH 7.4.

Cell culture and cell cycle analysis

Human NSCLC cell lines NCI-H1650 were obtained from the Chinese Academy of Science Cell Bank (Shanghai, China). NCI-H1650 was cultured in RPMI-1640 medium, with all recommended supplements. NCI-H1650 was maintained at 37 °C in a humidified incubator with 5% CO_2.

Cells were plated in 6-well plates at a density of 2×10^5 cells. After under serum starved condition, cells were treated with control, Nano-^{Mono}CPAP or Nano-^{Dimer}CPAP respectively. After 48 hours, the cells were separately collected, Washed twice with cold PBS, resuspended in 500 μL of PBS, added 4.5 mL of 70% ethanol while shaking and mixed, and fixed at -20 °C for 12 hours. After PBS washing, centrifugation, resuspending in 500 μL of 50 μg/mL ethidium bromide (PI), 100 ug/mL RNase A, 0.2% Triton X-100 in PBS, incubating at 4°C for 30 minutes in the dark, and using flow cytometry analysis. FlowJo software was used for analysis of cell cycle.

Transcriptome analyses

NCI-H1650 cells were seeded in 6-well plates and allowed to attach overnight. Cells were incubated with control, Nano-^{Mono}CPAP or Nano-^{Dimer}CPAP at a concentration of 2×10^5 particles/cell in RPMI-1640 medium with 10% FBS for 24 h at 37 °C. Total RNA from cells were isolated using Trizol and the quantity and quality of the resulting RNA was measured using a 2100 Bioanalyzer chip.

In vivo antitumor efficacy of Nano-^{Dimer}CPAP

Animal studies were performed according to the protocols approved by the Institution Guidelines and were approved by the Laboratory Animal Center of Xi'an Jiaotong University. ALL mice were purchased from the Laboratory Animal Center of Xi'an Jiaotong University. The mice were housed under standard specific pathogen-free conditions with a 12h–12h light–dark cycle.

Subcutaneous tumor-bearing mice model

C57BL/6 mice (aged 6–7 weeks) were age-matched for tumour inoculation. LLC cells (1 $\times10^6$ cells/site) were implanted subcutaneously into hip of C57BL/6 mice. When the tumors reached average volume of ~ 75 mm^3, mice were selected randomly into control group, Nano-^{Mono}CPAP (2.5mg/Kg) or Nano-^{Dimer}CPAP (2.5mg/Kg) group respectively (6 mices per group). Treatment was administered via intraperitoneal injection every other day. The body weight and condition of mice were monitored daily. In addition, tumor length and width were measured with calipers daily, and tumour volumes were calculated using the following equation: $1/2\times l\times w^2$. The humane end points were determined on the basis of the level of animal discomfort and tumour

sizes.

Patient-derived xenografts mice model

The homogenized tumor tissue with non-necrotic was cut into about 5 mm pieces and implanted into the right upper limb of each Female Balb/c nude mice (4–5 weeks) while under anesthesia. After two weeks, mice were selected into different groups. Then we treated tumor-burdened mice with PBS, Nano-^{Mono}CPAP (2.5mg/Kg) or Nano-^{Dimer}CPAP (2.5mg/Kg) through intravenous injection every other day, and monitored the tumor growth for 15 days. Daily monitoring of mice was the same as above subcutaneous tumor-bearing mice.

H&E and immunohistochemistry

All sections used for histological analysis were 4-μm thick. The tumors and the major organs (heart, liver, spleen, lung, and kidney) after different treatments were harvested for H&E staining. Moreover, the tumors were sectioned for cell apoptosis analysis by TUNEL apoptosis assay.

For immunohistochemistry, primary antibodies were used: anti-p53, Ki-67 (Proteintech, USA; 1: 200)， anti-MDMX, p73(Abcam, USA; 1: 100), MDM2 (Abcam, USA; 1: 200). Images of representative fields were taken with a Nikon Eclipse Ni-U microscope through the NIS Elements imaging software (Nikon) and quantified through ImageJ (NIH).

Statistics

All the experimental data were measured in triplicate at least and are presented as mean ± standard deviation unless otherwise mentioned. Statistical variance of two comparison groups was

performed at a significance level of $p < 0.05$ based on a Student's t-test. comparisons of more than two groups were calculated using a one-way analysis of variance (ANOVA), or log-rank test where necessary.

References

[1] Chen, X.; Gohain, N.; Zhan, C.; Lu, W.; Pazgier, M.; Lu, W. Structural Basis of How Stress-Induced MDMX Phosphorylation Activates p53. Oncogene 2016, 35, 1919.

[2] He, W.; Mazzuca, P.; Yuan, W.; Varney, K.; Bugatti, A.; Cagnotto, A.; Giagulli, C.; Rusnati, M.; Marsico, S.; Diomede, L.; Salmona, M.; Caruso, A.; Lu, W.; Caccuri, F. Identification of Amino Acid Residues Critical for the B Cell Growth-Promoting Activity of HIV-1 Matrix Protein p17 Variants. Biochim. Biophys. Acta, Gen. Subj. 2019, 1863, 13−34.

2. Statistical mechanics theory

Derivation of the dissociation constant based on statistical mechanics

Suppose that a ligand (*L*) can bind to a receptor (*R*). The dissociation constant K_d for the chemical reaction between receptors and ligands is defined as

$$K_d = \frac{[R][L]}{[RL]}, \tag{S1}$$

where $[RL]$ is the concentration of *R*-*L* bonds, $[R]$ and $[L]$ are the concentrations of free receptors and ligands, respectively. In the ideal limit, the partition functions Q_I (*I*=*R*, *L* and *RL*) of three species are

$$Q_R = \frac{(\mathrm{q}_R)^{N_R}}{N_R!}, \tag{S2}$$

$$Q_L = \frac{(\mathrm{q}_L)^{N_L}}{N_L!}, \tag{S3}$$

$$Q_{RL} = \frac{(\mathrm{q}_{RL})^{N_{RL}}}{N_{RL}!}, \tag{S4}$$

where q_I is the partition function of a single molecule of species *I*, and N_I is the number of the molecule *I*. The Hamiltonian of a free receptor or ligand is $H_u = E_k^{(u)} + U_f$ with $E_k^{(u)}$ being the kinetic energy and U_f being the potential of the molecule. The Hamiltonian of a *R*-*L* bond is $H_b = E_k^{(b)} + U_{RL}$, where $E_k^{(b)}$ is the kinetic energy and U_{RL} is the potential of the bond.

The partition function q_R or q_L of a free receptor or ligand involves three translational degrees of freedom (described by ***x***, ***y***, and ***z***) and three rotational degrees of freedom (described by θ, φ, and ϕ). The degrees of freedom of the partition function q_{RL} of a single *R*-*L* bond is the sum of those of q_R and q_L. Then, the partition functions q_I of a single molecule are

respectively

$$q_R = \frac{1}{h_p^6}\iiiint e^{-H_u/k_BT}\ d\vec{p}_r d\vec{p}_\Omega d\vec{r} d\vec{\Omega}, \tag{S5}$$

$$q_L = \frac{1}{h_p^6}\iiiint e^{-H_u/k_BT}\ d\vec{p}_r d\vec{p}_\Omega d\vec{r} d\vec{\Omega}, \tag{S6}$$

$$q_{RL} = \frac{1}{h_p^{12}}\iiiint e^{-H_b/k_BT}\ d\vec{p}_r d\vec{p}_\Omega d\vec{r} d\vec{\Omega}, \tag{S7}$$

where h_p is Planck's constant, $\vec{p}_r$ and $\vec{p}_\Omega$ are respectively linear and angular momenta, $\vec{r}$ and $\vec{\Omega}$ are the positions and orientations of the molecular domains. For the free molecule, integrations over the linear momenta in Eqs. (S5) and (S6) yield [1]

$$\iiint e^{-(P_x^2+P_y^2+P_z^2)/2m_Rk_BT}\ dP_x dP_y dP_z = \left(2\pi m_R k_B T\right)^{3/2}, \tag{S8}$$

$$\iiint e^{-(P_x^2+P_y^2+P_z^2)/2m_Lk_BT}\ dP_x dP_y dP_z = \left(2\pi m_L k_B T\right)^{3/2}, \tag{S9}$$

where m_R and m_L are the masses of receptor and ligand, respectively. Integrations over the angular momenta in Eqs. (S5) and (S6) yield [2]

$$\iiint e^{-(P_\theta^2/2I_{R,\theta}+P_\varphi^2/2I_{R,\varphi}+P_\phi^2/2I_{R,\phi})/k_BT}\ dP_\theta dP_\varphi dP_\phi = \left(2\pi k_B T\right)^{3/2}(I_{R,\theta}I_{R,\varphi}I_{R,\phi})^{1/2}\sin\theta, \tag{S10}$$

$$\iiint e^{-(P_\theta^2/2I_{L,\theta}+P_\varphi^2/2I_{L,\varphi}+P_\phi^2/2I_{L,\phi})/k_BT}\ dP_\theta dP_\varphi dP_\phi = \left(2\pi k_B T\right)^{3/2}(I_{L,\theta}I_{L,\varphi}I_{L,\phi})^{1/2}\sin\theta, \tag{S11}$$

where $I_{i,\theta}$, $I_{i,\varphi}$, and $I_{i,\phi}$ are three principal moments of inertial of the molecule *i*. The integral over the angular momenta space contributes a factor $\Gamma_R = \Gamma_L = \left(2\pi k_B T\right)^{3/2}(I_\theta I_\varphi I_\phi)^{1/2}$. The factor $\sin\theta$ will be taken into account when one further integrates with respect to the orientation coordinates in q_I. Then, the partition function of a free receptor or ligand becomes

$$q_R = \frac{\left(2\pi m_R k_B T\right)^{3/2}\left(2\pi k_B T\right)^{3/2}(I_\theta I_\varphi I_\phi)^{1/2}}{h_p^6} Z_R = \frac{1}{h_p^6}\left(2\pi m_R k_B T\right)^{3/2}\Gamma_R Z_R, \tag{S12}$$

$$q_L = \frac{\left(2\pi m_L k_B T\right)^{3/2}\left(2\pi k_B T\right)^{3/2}(I_\theta I_\varphi I_\phi)^{1/2}}{h_p^6} Z_L = \frac{1}{h_p^6}\left(2\pi m_L k_B T\right)^{3/2}\Gamma_L Z_L, \quad \text{(S13)}$$

where the configurational space Z_R or Z_L involve the integrals over the position and the orientation coordinates of a free receptor or ligand molecule. For a *R*-*L* bond, its kinetic energy is the sum of the contribution of *R* and that of *L* in the bond. The integral over the angular momenta space contributes a same factor $\Gamma_{RL} = \Gamma_R = \Gamma_L$, and the factor $\sin\theta$ will be included in the orientation integral. Then, the partition function of a bond is

$$q_{RL} = \frac{1}{h_p^{12}}(2\pi m_R k_B T)^{3/2}(2\pi m_L k_B T)^{3/2}\Gamma_R \Gamma_L Z_{RL}, \quad \text{(S14)}$$

where the configurational space Z_{RL} is an integral over the position and the orientation coordinates of a bond in the solution.

Using the partition functions defined in Eqs. (S2–S4), we can calculate the free energy

$$F_I = -k_B T \ln Q_I. \quad \text{(S15)}$$

Using the chemical potential

$$\mu_I = \partial F_I / \partial N_I \quad \text{(S16)}$$

and the equilibrium condition of the reaction

$$\mu_{RL} = \mu_R + \mu_L, \quad \text{(S17)}$$

one can obtain

$$\frac{N_R N_L}{N_{RL}} = \frac{Z_R Z_L}{Z_{RL}}. \quad \text{(S18)}$$

The concentration is defined as $[I] = N_I / V$, and then the dissociation constant in Eq. (S1) is

re-expressed as

$$K_d = \frac{(N_R/V)(N_L/V)}{N_{RL}/V} = \frac{Z_R Z_L}{Z_{RL} V}. \tag{S19}$$

Using Eqs. (S12)–(S14), the difference of the free energy between a bond, a free receptor and a free ligand is

$$\Delta G = -k_B T \ln\left(\frac{q_{RL}}{q_R q_L}\right) = -k_B T \ln\left(\frac{Z_{RL}}{Z_R Z_L}\right), \tag{S20}$$

where the terms related to the kinetic energy is cancelled. Equation (S20) indicates that the kinetic energy plays no role in ΔG, although it affects the absolute free energy of molecules. Substituting Eq. (S20) into Eq. (S19) leads to

$$K_d = \frac{1}{V} e^{\Delta G/k_B T}. \tag{S21}$$

The configurational integrals of an unbound receptor or ligand are respectively

$$Z_R = \int\int\int\int\int\int \sin\theta \, \mathrm{d}\theta \mathrm{d}\varphi \mathrm{d}\phi \mathrm{d}x \mathrm{d}y \mathrm{d}z = \Omega_R V, \tag{S22}$$

$$Z_L = \int\int\int\int\int\int \sin\theta \, \mathrm{d}\theta \mathrm{d}\varphi \mathrm{d}\phi \mathrm{d}x \mathrm{d}y \mathrm{d}z = \Omega_L V, \tag{S23}$$

where $\Omega_R = 8\pi^2$ and $\Omega_L = 8\pi^2$ represent the rotational phase-space volumes of a free receptor or ligand, respectively. For a *R-L* bond, the configurational integral is

$$Z_{RL} = \int ... \int e^{-U_b/k_B T} \sin\theta \; d\vec{r}_R d\vec{\Omega}_R d\vec{r}_{RL} d\vec{\Omega}_{RL} = \Omega_{RL} \Omega_b V V_b e^{-U_b/k_B T}, \tag{S24}$$

where U_b is the binding enthalpy of a receptor and a ligand, $\vec{r}_R$ and $\vec{\Omega}_R$ are respectively the spatial positions and rotational orientations of the receptor, $\vec{r}_{RL}$ and $\vec{\Omega}_{RL}$ are respectively the spatial positions and rotational orientations of the ligand with respect to the receptor in the bond, V_b is the free volume of the binding site of the ligand with respect to the binding site of the

receptor, Ω_{RL} is the rotational space volume of the receptor-ligand bond, and Ω_b is the rotational space volume of the binding site of the ligand with respect to the binding site of the receptor in the bond.

Using Eqs. (S19), (S22), (S23) and (S24), we can obtain

$$K_d = \frac{Z_R Z_L}{Z_{RL} V} = \frac{\Omega_R \Omega_L}{\Omega_{RL} \Omega_b V_b} e^{U_b / k_B T} . \tag{S25}$$

Using Eqs. (S20), (S22), (S23) and (S24), we get the free energy difference ΔG upon binding

$$\begin{aligned} \Delta G &= U_b - k_B T \ln\left(\frac{V_b}{V}\right) - k_B T \ln\left(\frac{\Omega_{RL}\Omega_b}{\Omega_R \Omega_L}\right) . \\ &= U_b - T\Delta S_{\text{trans}} - T\Delta S_{\text{rot}} \end{aligned} \tag{S26}$$

where $\Delta S_{\text{trans}} = -k_B \ln\left(V_b / V\right)$ represents the loss of translational entropy upon the binding, and $\Delta S_{\text{rot}} = -k_B \ln\left(\Omega_{RL}\Omega_b / \Omega_R \Omega_L\right)$ represents the loss of rotational entropy upon the binding. It should be noted that Eq. (S25) can be also obtained by substituting Eq. (S26) into Eq. (S21).

References

[3] T. L. Hill, *An Introduction to Statistical Thermodynamics* (Addison-Wesley, London, 1987).

[4] J. E. Mayer and M. G. Mayer, *Statistical Mechanics* (John Wiley, New York, 1940).

3. Supplementary figures

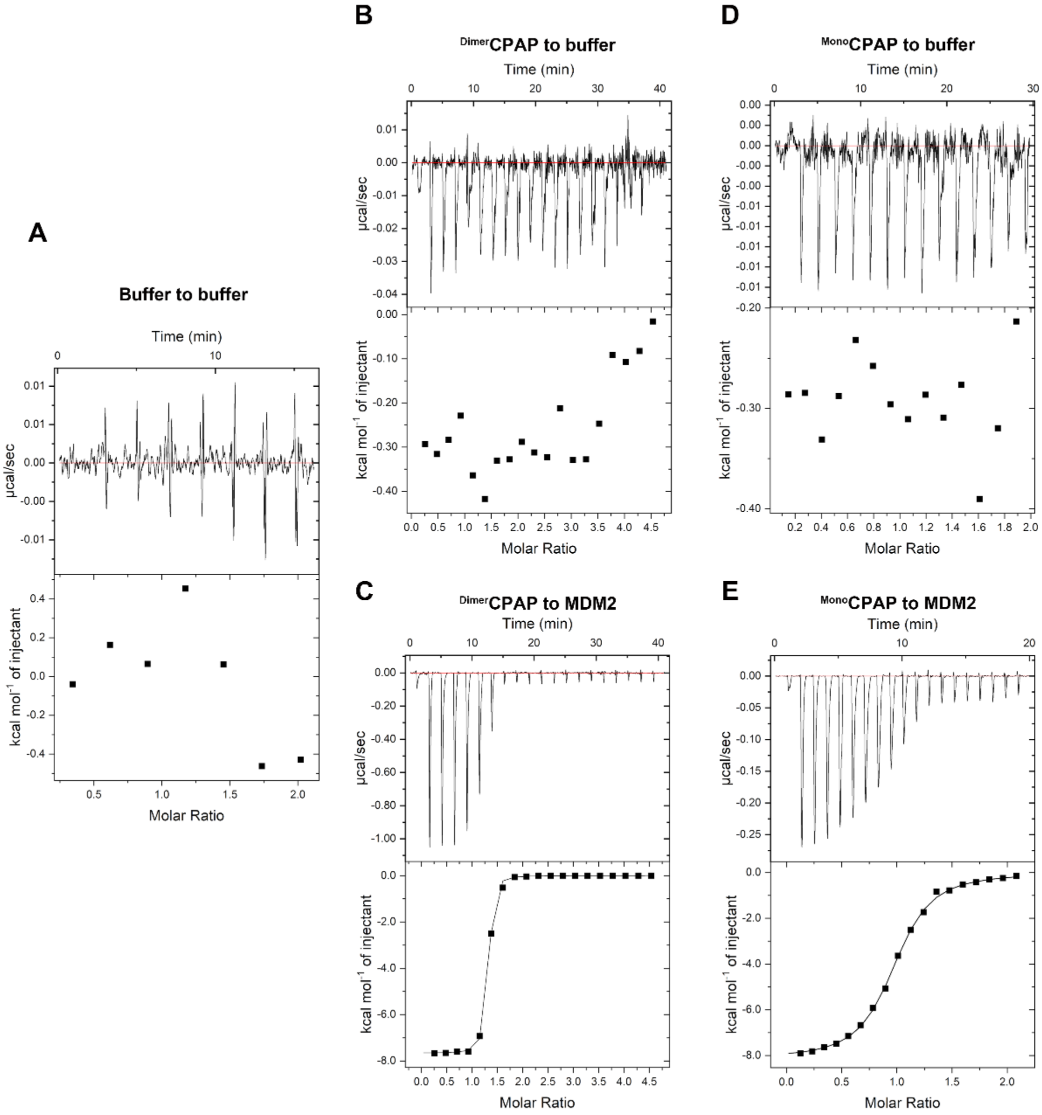

Figure S1. Quantification of the interactions of MDM2 or buffer with varying concentrations of ^{Dimer}CPAP and ^{Mono}CPAP by isothermal titration calorimetry (ITC). (A) Titrate PBS buffer with PBS buffer. (B) Titrate PBS buffer with ^{Dimer}CPAP in PBS buffer. (C) Titrate MDM2 in PBS buffer with ^{Dimer}CPAP in PBS buffer. (D) Titrate PBS buffer with ^{Mono}CPAP in PBS buffer. (E) Titrate MDM2 in PBS buffer with ^{Mono}CPAP in PBS buffer.

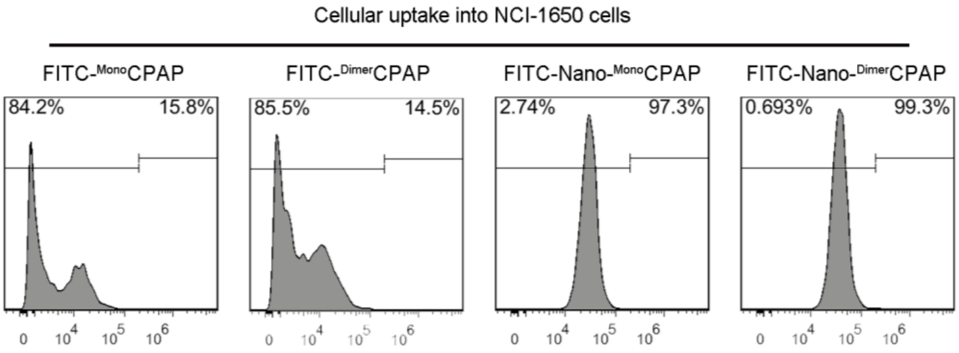

Figure S2. Cellular uptakes of FITC-labeled ^{Mono}CPAP, ^{Dimer}CPAP, Nano-^{Mono}CPAP and Nano-^{Dimer}CPAP into NCI-1650 lung adenocarcinoma cell measured by flow cytometry.

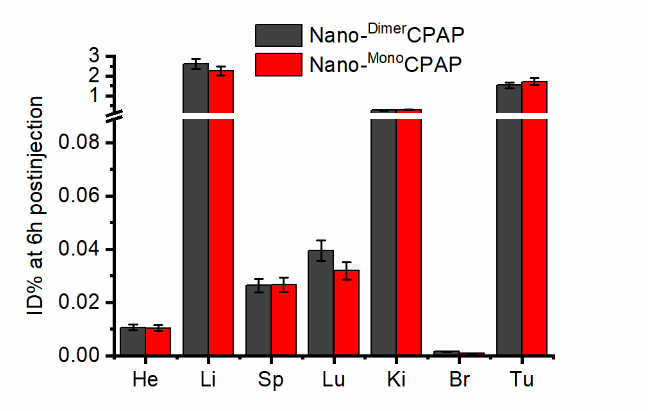

Figure S3. Biodistribution of Nano-^{Mono}CPAP and Nano-^{Dimer}CPAP in C57 mice bearing LLC cells iv. Dosing measured by ICP-MS.

\

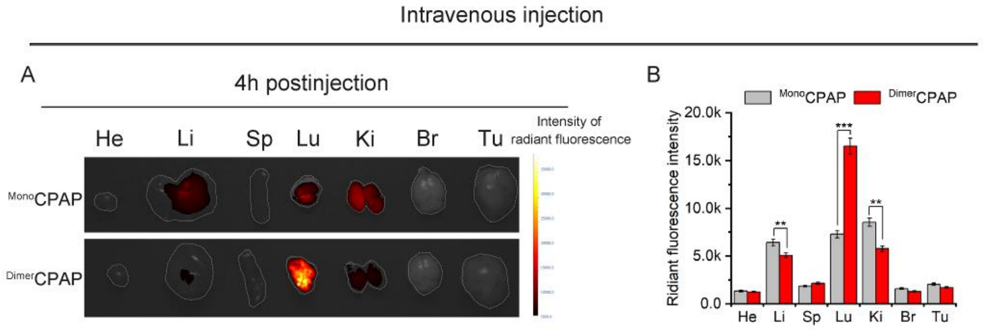

Figure S4. Biodistribution of ^{Mono}CPAP and ^{Dimer}CPAP in C57 mice bearing LLC cells iv measuring. **A**) representative ex vivo fluorescence image of major organs and tumor at 4 h after intravenous injection of A ^{Mono}CPAP and ^{Dimer}CPAP in mice. He: heart; Li: liver; Sp: spleen; Lu: lung; Ki: kidneys; Br: Brain; Tu: tumor. **B**) Ex vivo semi-quantitative analysis of biodistribution.

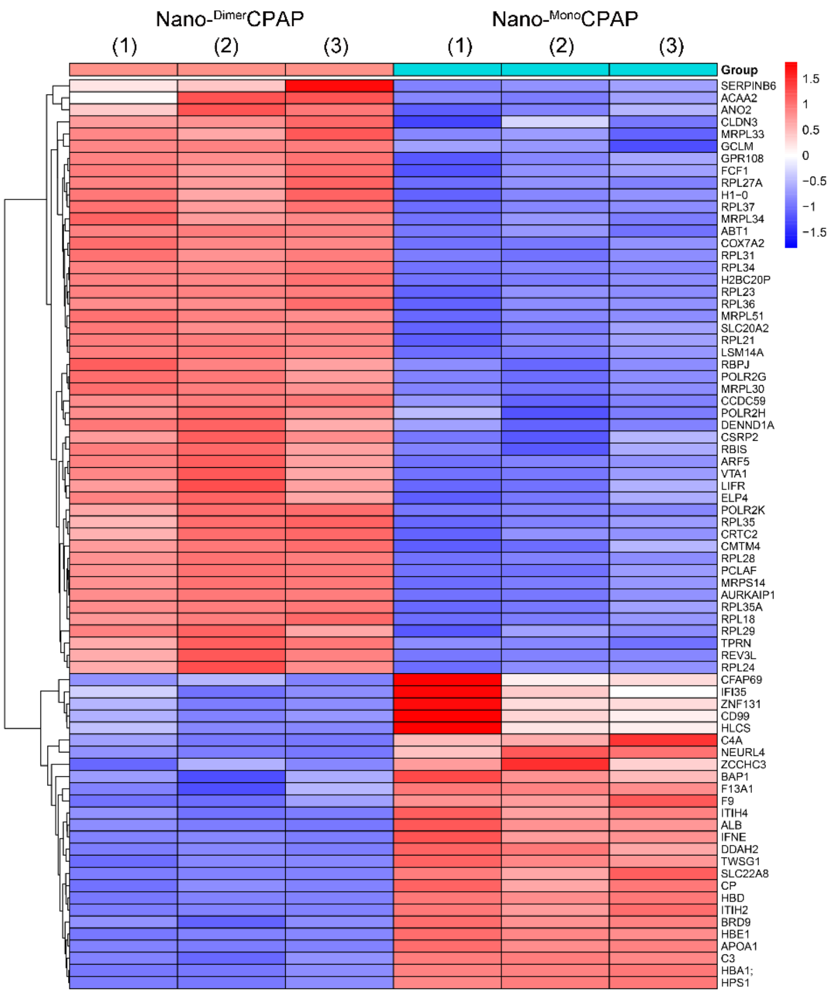

Figure S5. Hierarchical clustering of genes differentially expressed in NCI-H1650 cells after exposure to Nano-^{Dimer}CPAP or Nano-^{Mono}CPAP (n = 3).

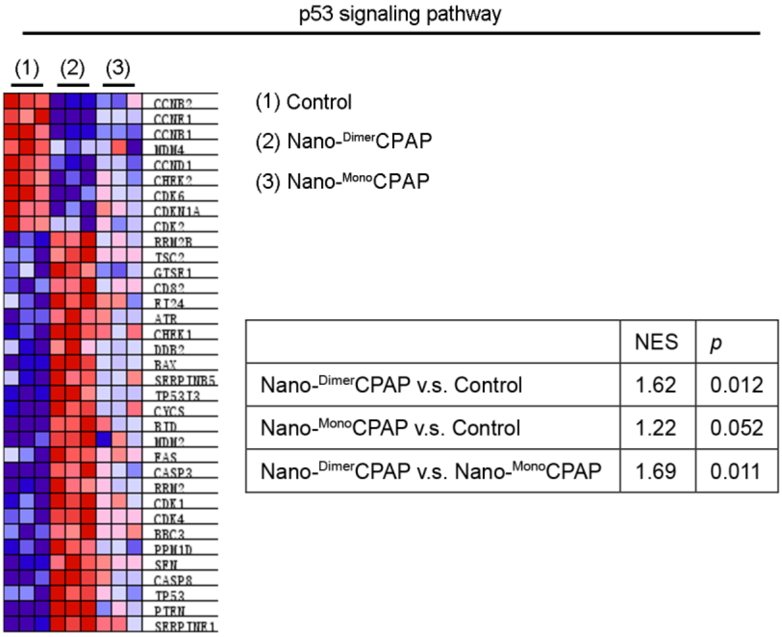

	NES	*p*
Nano-DimerCPAP v.s. Control	1.62	0.012
Nano-MonoCPAP v.s. Control	1.22	0.052
Nano-DimerCPAP v.s. Nano-MonoCPAP	1.69	0.011

Figure S6. Heat map of RNA-Seq analysis of NCI-H1650 cells' mRNAs which were differentially expressed. Control is the CtrlNano.

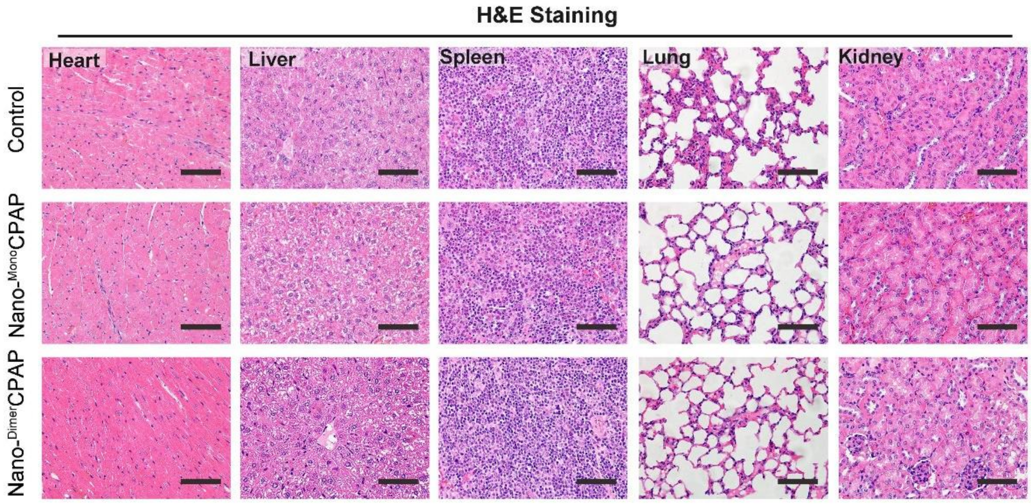

Figure S7. Toxicity evaluation of the visceral organ in mice with different treatments. The representative H&E staining of visceral tissues in mice with the indicated treatments (scale bar: 50 μm).

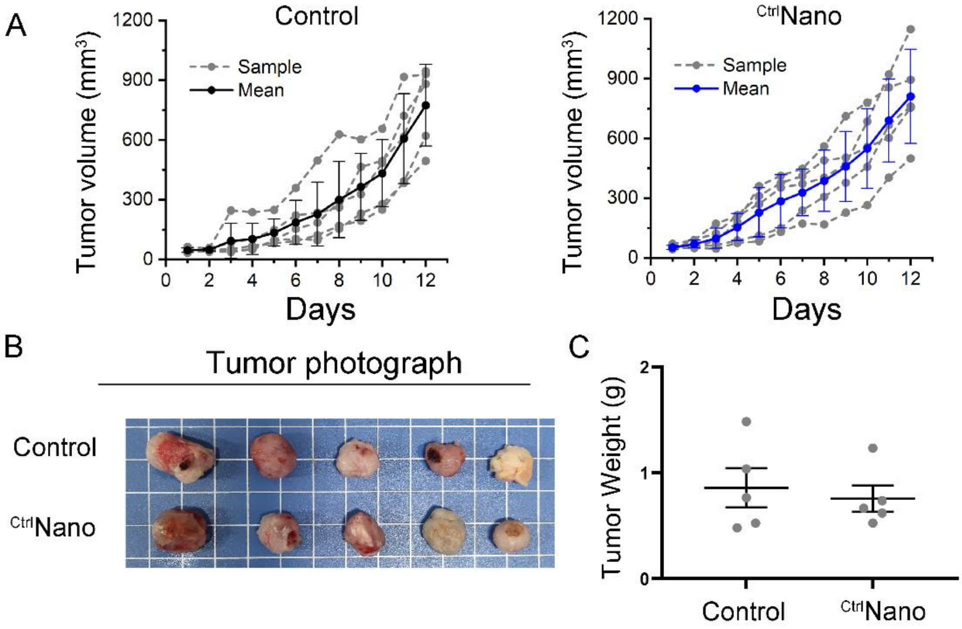

Figure S8. LLC homograft model in C57/B6 mice treated with Control and ^{Ctrl}Nano (n = 5, mean ± sd). **A**) Growth curves of LLC homograft model in C57/B6 mice with treatments, following the administration of control (PBS), ^{Ctrl}Nano. **B&C**) Representative photographs (B) and weight (C) of tumor tissue isolated at the end of experiment.

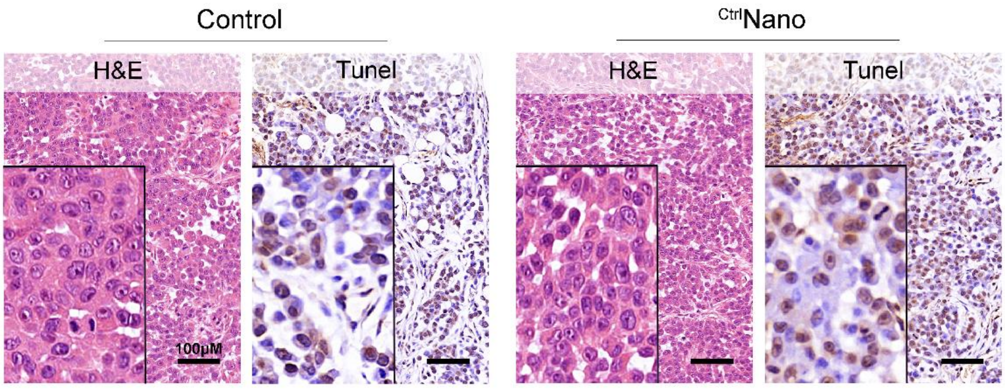

Figure S9. The H&E and TUNEL staining in tumor from mice treated with PBS and ^{Ctrl}Nano.

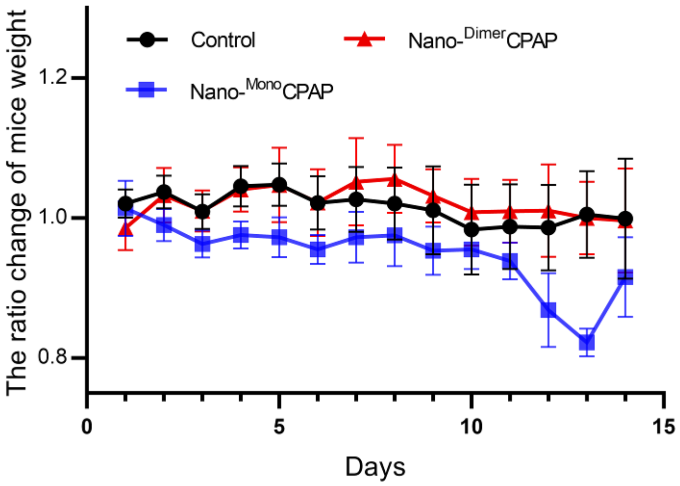

Figure S10. Weight of NOD/SCID mice bearing PDX tumor with the indicated treatments. The data were presented as mean ± s.d. of values from five independent experiments.

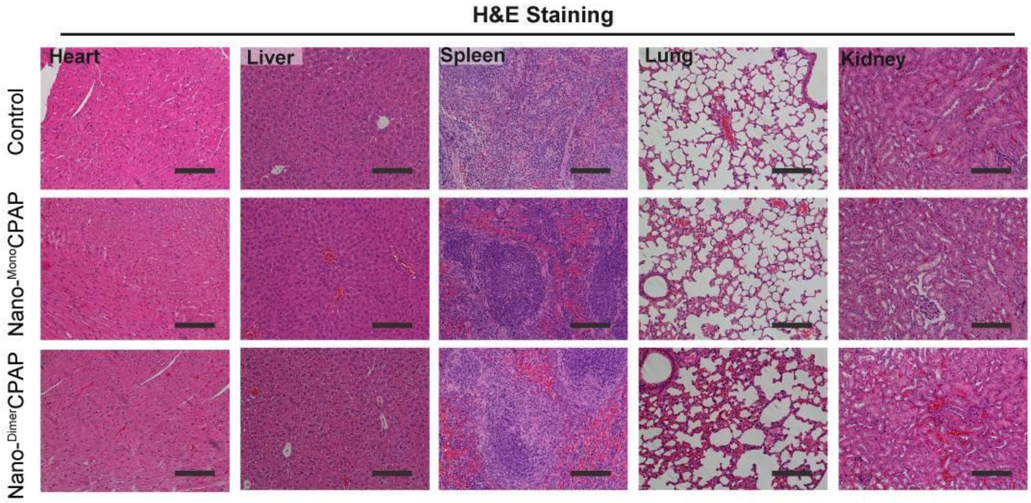

Figure S11. Toxicity evaluation of the visceral organ in Balb/c nude mice with different treatments. The representative H&E staining of visceral tissues in mice with the indicated treatments (scale bar: 100 μm).

4. Supplementary Table

Table S1. The value of cell cycle via flow cytometry

Cell Cycle	Control	^{Ctrl}Nano	Nano-^{Mono}CPAP	Nano-^{Dimer}CPAP
G0/G1	66.92%	68.9%	57.16%	56.69%
S	34.78%	28.15%	8.71%	16.98%
G2/M	0.3%	2.56%	32.96%	23.55%
